# Occlusion of functional high-volume intra-atrial shunts in older patients after embolic stroke of undetermined source

**DOI:** 10.3389/fcvm.2024.1402137

**Published:** 2024-09-27

**Authors:** Helene Schrader, Leif-Hendrik Boldt, Abdul S. Parwani, Florian Blaschke, Julia M. Wiedenhofer, Tobias D. Trippel, Gerhard Hindricks, Christoph Starck, Henryk Dreger, Mohammad Sherif, Uwe Primessnig

**Affiliations:** ^1^ Department of Cardiology, Angiology and Intensive Care Medicine, Deutsches Herzzentrum der Charité, Berlin, Germany; ^2^ Charité—Universitätsmedizin Berlin, Corporate Member of Freie Universität Berlin and Humboldt-Universität zu Berlin, Berlin, Germany; ^3^ DZHK (German Centre for Cardiovascular Research), Berlin, Germany; ^4^ Department of Cardiothoracic and Vascular Surgery, Deutsches Herzzentrum der Charité, Berlin, Germany

**Keywords:** patent foramen ovale (PFO), atrial septum defect (ASD), paradox embolism, transcatheter occlusion, embolic stroke of undetermined source (ESUS)

## Abstract

**Background:**

Intra-atrial shunts are associated with an elevated risk of embolic stroke of undetermined source (ESUS). Percutaneous occluder implantation is recommended as secondary prevention in younger patients. This study aims to compare the outcome after shunt occlusion between younger and older patients with a history of presumed paradox embolism and to evaluate the impact of high-volume shunting in an elderly population.

**Methods:**

We conducted a single-center, retrospective, observational study, involving 187 patients who underwent interventional percutaneous PFO or ASD occlusion at our center between 2013 and 2023.

**Results:**

The mean age of participants was 51.8 ± 11.8 years, with 76 patients aged ≤50 years and 111 patients aged >50 years. Older patients presented more cardiovascular risk factors. The presence of atrial septum aneurysm or large shunting was evenly distributed (ASA 26.3% vs. 28.8%, *p* = 0.833, mean shunt defect size 6.67 vs. 7.23 mm, *p* = 0.151). There were no significant differences in procedural or intrahospital complications. The event rate during the 6-month follow-up was low. Recurrence of arterial embolism occurred in 1.6% of the younger and 3.8% of the older patients (*p* = 0.817). Comparison of high-volume shunts (defect size ≥10 mm or passage ≥20 bubbles during bubble study) with low-volume shunts in this elderly cohort with a mean age ≥50 years showed no significant difference in outcomes. There was a statistically non-significant trend toward a higher rate of residual shunt at the end of the procedure in the high-volume shunt group (2.9% vs. 9.8%, *p* = 0.0894). This difference was not observed at the 6-month follow-up anymore (14.5 vs. 12.1%, *p* = 0.628). Two unsuccessful implantation attempts were reported in the high-volume shunt group, while none were observed in the low-volume shunt group (*p* = 0.372). No intervention-related deaths occurred in this patient cohort during follow-up.

**Conclusion:**

Occlusion of relevant, intra-atrial shunting is a safe and effective option for secondary prevention of cryptogenic embolism in patients over 50 years of age. The beneficial outcome was irrespective of a high-volume shunting before implantation.

## Introduction

Patent foramen ovale (PFO) and atrial septum defects (ASD) are associated with an elevated risk of embolic stroke of undetermined source (ESUS) (1). ESUS represents approximately 30% of all strokes, and one-third of the affected patients will experience a recurrence within the next 10 years (2), which emphasizes the need for a deliberate concept regarding secondary prophylaxis. Given the high prevalence of PFO and ASD in patients with ESUS, it is assumed that intra-atrial shunting promotes paradox embolism through thrombus transfer from venous to arterial circulation by right-to-left shunting (3). Consequently, guidelines suggest percutaneous occlusion of intra-atrial shunts in selected patients. In these patients, large, controlled trials have demonstrated an effective prevention of recurrent stroke in comparison to antiplatelet therapy (4–10). Due to PFO being the most common congenital cardiac defect affecting approximately 25% of the general adult population (11), criteria for patient selection are important, to differentiate random findings from causative shunts. The established criteria for patient selection include echocardiographic factors such as defect size and length and the presence of an atrial septum aneurysm (ASA) and clinical risk factors, such as patient age, high likelihood for non-PFO-associated stroke mechanisms (e.g., advanced atherosclerosis) and infarct location (12). Most large randomized controlled trials (RCTs) enrolled only patients aged over 60 years. Current guideline recommendations therefore recommend closure of IAS in patients with ESUS only in patients who are younger than 60 years. However, the association between intra-atrial shunting and ESUS has also been shown in elderly patient groups (1, 3, 13). This study therefore aims to compare the outcome of adult patients with a history of ESUS, transient ischemic attack (TIA), or central retinal artery occlusion (CRAO), who underwent percutaneous occlusion of an intra-atrial shunt, depending on their age group. Furthermore, we seek to answer the question of whether the outcome in a generally elderly population is influenced by functional high-risk features such as the presence of a high-volume shunt (HVS).

## Methods

We conducted a single-center, retrospective, observational study involving 187 patients who underwent interventional percutaneous occlusion of an intra-atrial shunt at the adult cardiology department of the Charité—Universitätsmedizin Berlin Campus Virchow between February 2013 and February 2023. Eligible patients were adults over 18 years; had a history of ESUS, TIA, or CRAO; and had an echocardiographic proof of a relevant right-to-left shunting via PFO, ASD secundum type, or complex septal defects. In all patients, the ischemic event was diagnosed by neurologists or ophthalmologists according to standard clinical practice. After evaluation of further risk factors and comorbidities, paradox embolism was considered the most likely explanation for the defining ischemic event by the involved physicians. Diagnostics performed to obtain this conclusion included cerebral imaging with either brain MRI or CT, ophthalmoscopy in patients with CRAO, polysomnography in selected patients with high risk for OSAS, long-term Holter ECG, carotid sonography, and echocardiography.

In all patients, transthoracic and transoesophageal echocardiography (TEE) were performed before and during the intervention and again 3–6 months after the intervention. Shunt size was quantified by bubble study after injection of agitated saline contrast medium with a Valsalva maneuver or abdominal compression to increase right atrial pressure. The presence of ASA was identified by either additional echocardiographic measurements or by intraprocedural balloon sizing. ASA was defined by a protrusion of the atrial septum into the right or left atrium of at least 15 mm with a base diameter of at least 15 mm (14). PFO size was divided into three groups by the amount of bubbles passing after three successive cardiac cycles under theValsalva maneuver during the echocardiographic bubble study: 1–4 bubbles were categorized as “grade 1,” 5–20 bubbles as “grade 2,” and >20 bubbles as “grade 3” (15). An HVS was defined as a defect size ≥10 mm or >20 bubbles passing during bubble study (grade 3).

The procedure was performed according to current clinical standards with transoesophageal and fluoroscopic guiding. The choice of device type and size was made according to anatomical features and the experience of the interventionalist. A successful implantation was defined as a stable positioning of the occluder in the intra-atrial septum without major protrusion and the absence of residual peri-device shunting.

Immediately after the procedure and again before hospital discharge, patients received transthoracic echocardiography to control device position and exclude pericardial effusion. During the 6-month follow-up (FU), device position, residual shunting, and the evaluation of thrombus formation on the occluder surface were carried out by transesophageal echocardiography. All patients received either dual antiplatelet therapy or continued pre-existing oral anticoagulation until FU TEE. Analysis at 6-month follow-up represents a landmark analysis of the hospital survivors and only included patients in the evaluation, who participated in the 6-month FU TEE. Extended follow-up beyond this time point was limited to a retrospective review of subsequent hospital admissions within our institution.

The study was approved by the local ethical committee from the Charité (EA4/031/24). Clinical data were extracted retrospectively from electronic medical records. Complete case analysis was performed for all data points. All analyses were performed on pseudonymized datasets to protect patient privacy and confidentiality.

### Statistical analysis

Statistical analyses were performed using R 4.2.3 (R Core Team 2023). For continuous variables, normality was assessed using the Shapiro–Wilk test. The continuous variables are reported as means (±standard deviations) if normally distributed or as medians [interquartile ranges] (IQR) if not normally distributed. The categorical variables are presented as absolute numbers and percentages. Student's *t*-test or Wilcoxon–Mann–Whitney *U*-test was employed for continuous variables, while the Chi-square test was used for categorical variables. A *P*-value of <0.05 was considered statistically significant.

## Results

### Study population by age groups

Our study included 187 patients who underwent percutaneous PFO or ASD occluder implantation at our institution between 2013 and 2023. The mean age of the participants was 51.8 ± 11.8 years. Seventy-six patients aged ≤50 years, and 111 patients aged>50 years. Our oldest patient was 81 years old, and 63% were male. In 94.1% of the patients, a PFO was present, and 9.1% showed an ASD. Patients in the elderly group had significantly more cardiovascular comorbidities such as arterial hypertension, coronary artery disease, hyperlipidemia, diabetes mellitus, or renal insufficiency. Furthermore, the group of patients aged >50 years had more atrioventricular blockage documented via ECG, while the presence of atrial fibrillation prior to implantation (1 patient vs. 5 patients, *p* = 0.428) was not different. In addition, 96.7% of the patients had a normal left ventricular ejection fraction (LVEF) over 50%, and 1.7% had a reduced LVEF under 40%. Elderly patients had a bigger size of the left atrium (LAVI 25.7 ± 5.73 ml/m^2^ in patients aged ≤50 years and 29.9 ± 7.58 ml/m^2^ in patients aged >50 years, *p* < 0.001). There was a statistically non-significant trend toward bigger defect sizes (6.67 ± 4.67 mm vs. 7.23 ± 4.0 mm, *p* = 0.151) and a higher right-to-left shunt volume (21.2 ± 24.9% vs. 25.2 ± 31.9%, *p* = 0.0944) in the elderly patients. There was no difference in the rate of HVS (40.8% vs. 45.9%, *p* = 0.584) or ASA (26.3% vs. 28.8%, *p* = 0.833). Other baseline criteria were balanced. Especially the number of patients with previous venous thromboembolism (17.1% vs. 13.5%, *p* = 0.64), coagulation disorders (7.9% vs. 3.6%, *p* = 0.342), or the preprocedural anticoagulation regimen did not differ between younger and older patients. The baseline characteristics of the study population are summarized in Tables 1A,B.

**Table 1A:** Clinical baseline parameters.

*N*	Age category				Shunt volume category			
	<50 years	>50 years	Total	*p*-Value	LVS	HVS	Total	*p*-value
	76	111	187		105	82	187	
Male	44 (57.9%)	74 (66.7%)	118 (63.1%)	0.286	65 (61.9%)	53 (64.6%)	118 (63.1%)	0.817
Comorbidities								
Atrial fibrillation	1 (1.3%)	5 (4.5%)	6 (3.2%)	0.428	1 (1.0%)	5 (6.1%)	6 (3.2%)	0.118
Arterial hypertension	14 (18.4%)	48 (43.2%)	62 (33.2%)	<0.001	34 (32.4%)	28 (34.1%)	62 (33.2%)	0.922
Coronary artery disease	0 (0%)	9 (8.1%)	9 (4.8%)	0.028	6 (5.7%)	3 (3.7%)	9 (4.8%)	0.759
Obesity	8 (10.5%)	7 (6.3%)	15 (8.0%)	0.442	8 (7.6%)	7 (8.5%)	15 (8.0%)	1
Myocardial infarct	0 (0%)	6 (5.4%)	6 (3.2%)	0.101	4 (3.8%)	2 (2.4%)	6 (3.2%)	0.913
Venous thromboembolic event (deep vein thrombosis, PE)	13 (17.1%)	15 (13.5%)	28 (15.0%)	0.64	18 (17.1%)	10 (12.2%)	28 (15.0%)	0.463
Coagulation disorder	6 (7.9%)	4 (3.6%)	10 (5.3%)	0.342	7 (6.7%)	3 (3.7%)	10 (5.3%)	0.562
Hyperlipidemia	15 (19.7%)	45 (40.5%)	60 (32.1%)	0.005	33 (31.4%)	27 (32.9%)	60 (32.1%)	0.952
Diabetes mellitus	1 (1.3%)	13 (11.7%)	14 (7.5%)	0.018	9 (8.6%)	5 (6.1%)	14 (7.5%)	0.72
Anticoagulation before implantation—no./total no. (%)								
ASA	49/76 (64.5%)	73/108 (67.6%)	122/184 (66.3%)	0.613	49/102 (48.0%)	73/82 (89.1%)	122/184 (66.3%)	0.682
DAPT	3/76 (4.0%)	1/108 (0.9%)	4/184 (2.2%)	0.708	3/102 (2.9%)	1/82 (1.2%)	4/184 (2.2%)	0.456
DOAK	8/76 (10.5%)	19/108 (17.6%)	27/184 (14.6%)	0.356	8/102 (7.8%)	19/82 (23.2%)	27/184 (14.6%)	0.812
VKA	3/76 (3.9%)	5/108 (4.6%)	8/184 (4.3%)	1	3/102 (2.9%)	5/82 (6.1%)	8/184 (4.3%)	1

**Table 1B:** Echocardiographic baseline parameters.

*N*	Age category				Shunt volume category			
	<50 years	>50	Total	*p*-value	LVS	HVS	Total	*p*-value
	76	111	187		105	82	187	
LVEF (%)								
—no./total no. (%)								
>50	74/75 (98.7%)	102/107 (95.3%)	176/182 (96.7%)	1	100/104 (96.2%)	76/78 (97.4%)	176/182 (96.7%)	1
41–49	1/75 (1.3%)	2/107 (1.9%)	3/182 (1.7%)	1/104 (1.0%)	2/78 (2.6%)	3/182 (1.7%)		
<40	0/75 (0%)	3/107 (2.8%)	3/182 (1.7%)	3/104 (2.9%)	0	(0%)	3/182 (1.7%)	
Shunt grade								
1	32 (42.1%)	35 (31.5%)	67 (35.8%)	0.323	66 (62.9%)	1 (1.2%)	67 (35.8%)	<0.001
2	18 (23.7%)	33 (29.7%)	51 (27.3%)		39 (37.1%)	12 (14.6%)	51 (27.3%)	
3	26 (34.2%)	43 (38.7%)	69 (36.9%)		0 (0%)	69 (84.1%)	69 (36.9%)	
Maximum shunt defect (mm)	6.67 (4.67)	7.23 (4.00)	7.01 (4.28)	0.151	4.44 (2.17)	10.3 (4.07)	7.01 (4.28)	<0.001
Fraction R–L shunting (%)	21.2 (24.9)	25.2 (31.9)	23.6 (29.3)	0.0944	8.71 (4.42)	42.6 (35.9)	23.6 (29.3)	<0.001
LAVI (ml/m²)	25.7 (5.73)	29.9 (7.58)	28.1 (7.16)	<0.001	26.9 (6.76)	29.7 (7.41)	28.1 (7.16)	0.004
PFO	71 (93.4%)	105 (94.6%)	176 (94.1%)	0.985	103 (98.1%)	73 (89.0%)	176 (94.1%)	0.021
ASD secundum type	10 (13.2%)	7 (6.3%)	17 (9.1%)	0.18	3 (2.9%)	14 (17.1%)	17 (9.1%)	0.001
Atrial septal aneurysm	20 (26.3%)	32 (28.8%)	52 (27.8%)	0.833	27 (25.7%)	25 (30.5%)	52 (27.8%)	0.577
HVS	31 (40.8%)	51 (45.9%)	82 (43.9%)	0.584	0 (0%)	82 (100%)	82 (43.9%)	ns

### Procedural parameters

In one patient of each age group, successful occluder implantation could not be achieved. Intraprocedural change of the device size or type was needed in 5.3% vs. 4.5% (*p* = 1). In the younger age group, an Amplatzer Occluder was used in 43.4% of the cases, Gore Septal Occluder in 34.2%, LifeTech CeraFlex Occluder in 18.4%, and NitOcclud PFO Occluder in 2.6%. In the elderly age group, 57.7% received an Amplatzer Occluder, 21.6% a Gore Septal Occluder, 19.8% a LifeTech CeraFlex Occluder, and no one a NitOcclud PFO Occluder. The mean device size was 25.4 ± 3.47 mm in the younger group and 25.3 ± 3.93 mm in the older group (*p* = 0.71). Procedure duration was similar (38.5 ± 21.6 min vs. 37.7 ± 28.7 min, *p* = 0.283). In the echocardiographic control at the end of the intervention, the rate of remaining intra-atrial shunting was comparable in both groups (5.3% vs. 6.3%, *p* = 1). No relevant device protrusion was observed in any patient. Two patients in the younger age group and three elderly patients showed minor pericardial effusion without the need for drainage, immediately after intervention. In one patient in the younger age group, an air embolism with ST-elevation occurred. The patient completely recovered without any long-term consequences. An overview of the procedural details is listed in Table 2.

**Table 2:** Procedural and intrahospital outcome.

*N*	Age category				Shunt volume category			
	<50	>50	Total	*p*-value	LVS	HVS	Total	*p*-value
	76	111	187		105	82	187	
Days in-hospital	2.00 [1.00, 53.0]	2.00 [1.00, 50.0]	2.00 [1.00, 53.0]	0.3	2.00 [1.00, 53.0]	2.00 [1.00, 14.0]	2.00 [1.00, 53.0]	0.374
Procedure duration (min)	38.5 (21.6)	37.7 (28.7)	38.0 (25.9)	0.283	36.2 (23.5)	40.3 (28.8)	38.0 (25.9)	0.369
Device finally implanted								
Amplatzer Occluder	33 (43.4%)	64 (57.7%)	97 (51.9%)	0.06	60 (57.1%)	37 (45.1%)	97 (51.9%)	0.177
Gore septal occluder	26 (34.2%)		24 (21.6%)	50 (26.7%)	23 (21.9%)	27 (32.9%)		50 (26.7%)
LifeTech CeraFlex Occluder	14 (18.4%)		22 (19.8%)	36 (19.3%)	20 (19.0%)	16 (19.5%)		36 (19.3%)
NitOcclud PFO occluder	2 (2.6%)		0 (0%)	2 (1.1%)	2 (1.9%)	0 (0%)		2 (1.1%)
Unsuccessful implantation attempt	1 (1.3%)	1 (0.9%)	2 (1.1%)	1	0 (0%)	2 (2.4%)	2 (1.1%)	0.372
Intraprocedural repositioning	4 (5.3%)	5 (4.5%)	9 (4.8%)	1	5 (4.8%)	4 (4.9%)	9 (4.8%)	1
Final device size (mm)	25.4 (3.47)	25.3 (3.93)	25.4 (3.74)	0.71	24.9 (3.42)	26.0 (4.05)	25.4 (3.74)	0.0229
Protrusion ≤10 mm	0 (0%)	0 (0%)	0 (0%)	1	0 (0%)	0 (0%)	0 (%)	1
Residual shunt	4 (5.3%)	7 (6.3%)	11 (5.9%)	1	3 (2.9%)	8 (9.8%)	11 (5.9%)	0.0894
Complications								
In-hospital death	1 (1.3%)	1 (0.9%)	2 (1.1%)	1	2 (1.9%)	0 (0%)	2 (1.1%)	0.589
Air embolism	1 (1.3%)	0 (0%)	1 (0.55%)	1	1 (1%)	0 (0%)	1 (0.5%)	0.349
Bleeding	0 (0%)	3 (2.7%)	3 (1.6%)	0.394	0 (0%)	3 (3.7%)	3 (1.6%)	0.165
Stroke	0 (0%)	0 (0%)	0 (0%)	NaN	0 (0%)	0 (0%)	0 (0%)	NaN
Pericardial effusion	1 (1.3%)	4 (3.6%)	5 (2.7%)	0.623	2 (1.9%)	3 (3.7%)	5 (2.7%)	0.779
Pericardial tamponade	0 (0%)	1 (0.9%)	1 (0.5%)	1	0 (0%)	1 (1.2%)	1 (0.5%)	0.896
Access site complication	1 (1.3%)	4 (3.6%)	5 (2.7%)	0.623	2 (1.9%)	3 (3.7%)	5 (2.7%)	0.779
Anticoagulation after implantation								
ASA	63 (82.9%)	93 (83.8%)	156 (83.4%)	1	91 (86.7%)	65 (79.3%)	156 (83.4%)	0.249
DAPT	60 (78.9%)	91 (82.0%)	151 (80.7%)	0.743	89 (84.8%)	62 (75.6%)	151 (80.7%)	0.165
DOAK	11 (14.5%)	14 (12.6%)	25 (13.4%)	0.882	11 (10.5%)	14 (17.1%)	25 (13.4%)	0.272
VKA	1 (1.3%)	4 (3.6%)	5 (2.7%)	0.617	2 (1.9%)	3 (3.7%)	5 (2.7%)	0.768

### Postprocedural outcome and in-hospital complications

There was one intrahospital death in both groups, both due to septic multi-organ failure and without connection to the occluder implantation. There were three cases of bleeding in the elderly patient group and none in the younger group (*p* = 0.394) between implantation and hospital discharge. One patient had gastrointestinal bleeding after transesophageal echocardiography, ultimately requiring endoscopic therapy. One patient experienced bleeding on the access site requiring interventional treatment and one patient developed epistaxis requiring conservative bleeding management. No patient required a transfusion. Access site complications were reported in one younger patient and in four elderly patients (1.3% vs. 3.6%, *p* = 0.623). One patient in the younger group and four patients in the elderly group developed pericardial effusion during the short-term hospital stay. One elderly patient needed interventional drainage due to the pericardial tamponade. Other complications included anemia without signs of bleeding and pneumonia. Anticoagulation at discharge varied. In addition, 80.7% of all patients received dual antiplatelet therapy at hospital discharge, 13.4% received direct oral anticoagulants, and 2.7% vitamin K antagonists. The detailed postprocedural outcomes are also shown in Table 2.

### Six-month follow-up

Six-month follow-up data were available for 75.9% of the patients (Table 3). Echocardiographic proof of residual shunting was observed in 16.1% of the younger patients and 11.3% in the elderly group (*p* = 0.425). No pericardial effusions or thrombus formation on the devices was observed at FU TEE. Recurrence of arterial thromboembolic events, such as TIA, stroke, or CRAO, occurred in one younger patient and three elderly patients (1.6% vs. 3.8%, *p* = 0.817). Three older patients reported bleeding and anemia since implantation (0 vs. 3.8%, *p* = 0.349). No cardiac decompensation or deaths until during the 6-month follow-up.

**Table 3:** Six-month follow-up.

***N***	Age category				Shunt volume category			
	<50	>50	Total	*p*-value	LVS	HVS	Total	*p*-value
	76	111	187		105	82	187	
Follow-up compliance	62 (81.6%)	80 (72.1%)	142 (75.9%)	1	76 (72.4%)	66 (80.5%)	142 (75.9%)	0.295
Thrombus formation on device no./total no. (%)	0/62 (0%)	0/80 (0%)	0/142 (0%)	NaN	0/76 (0%)	0/66 (0%)	0/142 (0%)	NaN
Residual shunt—no./total no. (%)	10/62 (16.1%)	9/80 (11.3%)	19/142 (13.4%)	0.425	11/76 (14.5%)	8/66 (12.1%)	19/142 (13.4%)	0.628
Pericardial effusion—no./total no. (%)	0/62 (0%)	0/80 (0%)	0/142 (0%)	NaN	0/76 (0%)	0/66 (0%)	0/142 (0%)	NaN
Bleeding or anemia—no./total no. (%)	0/62 (0%)	3/80 (3.8%)	3/142 (2.1%)	0.349	1/76 (1.3%)	2/66 (3.0%)	3/142 (2.1%)	0.884
TIA, stroke, or CRAO—no./total no. (%)	1/62 (1.6%)	3/80 (3.8%)	4/142 (2.8%)	0.817	2/76 (2.6%)	2/66 (3.0%)	4/142 (2.8%)	1
Cardiac decompensation—no./total no. (%)	0/62 (0%)	0 (0%)	0 (0%)	NaN	0/76 (0%)	0/66 (0%)	0/142 (0%)	NaN
Death—no./total no. (%)	0/62 (0%)	0 (0%)	0 (0%)	NaN	0/76 (0%)	0/66 (0%)	0/142 (0%)	NaN

### Long-term follow-up

Hospital admissions were analyzed up to 8 years post-intervention, with a mean FU duration of 21.1 ± 30.7 months. There was one death in the older age group due to end-stage cancer 7 months after the intervention. One device-related thrombus was detected in each group, and a recurrence of an arterial thromboembolic event occurred in one patient in the younger group and five patients in the older age group. Hospitalization due to new-onset atrial fibrillation was necessary in two younger patients and four older patients. Bleedings occurred in one patient of the young age group and three patients of the older age group. Details are shown in Table 4.

**Table 4:** Long-term follow-up (>6 months).

*N*	Age category			Shunt volume category		
	<50	>50	Total	LVS	HVS	Total
	76	111	187	105	82	187
Data availability, *n* (%)	22 (29.0%)	29 (26.1%)	51 (27.3%)	26 (24.8%)	25 (30.5%)	51 (27.3%)
Mean FU duration (in months)	25.6 (32.9)	17.8 (29.0)	21.1 (30.7)	22.3 (35.0)	18.3 (26.6)	21.1 (30.7)
Thrombus formation on device—reported cases	1	1	2	2	0	2
Bleeding or anemia—reported cases	1	3	4	2	2	4
TIA, stroke, or CRAO—reported cases	1	5	6	4	2	6
Atrial fibrillation—reported cases	2	4	6	3	3	6
Cardiac decompensation—reported cases	0	1	1	0	1	1
Death—reported cases	0	1	1	0	1	1

### High-volume shunts

In this population, with a mean age of 51.8 ± 11.8 years, we also formed subgroups to estimate the impact of a high-volume shunt on the outcome. There were 105 patients without criteria for HVS and 82 with an HVS. Shunt defect size was 4.44 ± 2.1 mm in the LVS group and 10.3 ± 4.1 mm in the HVS group (*p* < 0.001). The fraction of the shunting toward right-to-left shunting was 8.71 ± 4.42% in the LVS group and 42.6 ± 35.9% in the HSV group (*p* < 0.001). In addition, 62.9% of the LVS shunts could be graded as grade 1, 37.1% as grade 2, and none as grade 3, while in the HVS group, 1.2% could be graded as grade 1, 14.6% as grade 2, and 84.1% as grade 3 (*p* < 0.001). The clinical baseline criteria in both groups showed no significant differences (Table 1A). Echocardiographic parameters showed larger left atrial size in the HVS group (LAVI 26.9 ± 6.79 ml/m^2^ for the LVS vs. 29.7 ± 7.41 ml/m^2^, *p* = 0.004) and more patients with ASD or complex defects, than in the LVS group. There were no differences regarding LVEF or the presence of an ASA (Table 1B). In the HVS group, larger devices had to be used (24.9 ± 3.42 mm vs. 26.0 ± 4.05 mm, *p* = 0.0229). There was no difference in the used device types (Table 2). In two patients of the HSV group, the IAS could not be successfully closed, while no unsuccessful implantation attempt occurred in the LVS group (*p* = 0.372). There were no differences in procedure duration (36.2 ± 23.5 min vs. 40.3 ± 28.8 min, *p* = 0.369) or the rate of residual shunts (2.9% vs. 9.8%, *p* = 0.0894) (Table 2). Follow-up after implantation was comparable between both groups and was not influenced by shunt size (Tables 2, 3).

## Discussion

In this retrospective study, we observed that in patients over the age of 50 years, percutaneous occlusion of an intra-atrial right-to-left shunt is a safe option with high success, few adverse events, and a low recurrence rate of arterial embolism. We also found that in an elderly population with a mean age of 51.8 years, the absence of an HVS has no impact on a favorable outcome with a low event rate.

While large RCTs (5, 7–10) and observational studies (16–19) have established percutaneous occlusion of intra-atrial shunts as the standard of care for secondary prevention of ESUS in younger patients below the age of 60 years, data and recommendations for elderly patients are limited. An association between intra-atrial shunting and ESUS has also been described in this patient group (1, 3, 13). Furthermore, occlusion of intra-atrial shunts has been proven as a safe and feasible option even in elderly patients (20).

Our study included all patients with a history of cryptogenic thromboembolic events (stroke, TIA, CRAO) and echocardiographic proof of a relevant right-to-left shunting with a high likelihood for causality between the intra-atrial shunt and the ischemic event. Our study population included 9.1% of patients with an ASD and/or another complex defect (e.g., combined PFO and ASD). With a mean age of 51.8 years, our study population was older than the population in most RCTs evaluating secondary prophylaxis of arterial thromboembolism by PFO occlusion (5–10) with a mean age between 42.9 (5) and 46.3 (7) years in the occlusion groups. Yet, one RCT, the DEFENSE-PFO trial, included patients up to the age of 80 years, with a mean age in the study population of 49.3 years (21). Our population however represents the typical age range of corresponding observational studies of PFO closure, thereby reflecting the “real-world” situation outside of RCTs (22–26). The total mean age in these studies was 50.1 years for a total of 2,592 patients reported. The inclusion criteria were similar to our study with the difference, that ASD was excluded in all of the other studies. Similar to our patient cohort, a higher prevalence of cardiovascular comorbidities was observed in the elderly patient groups (22, 23, 25, 26).

### Atrial septum aneurysm and functional high-risk parameters

With an association of higher age to risk features, such as a larger defect size or higher prevalence of ASA (6), Alperi et al., Luermann et al., and Spies et al. reported populations with a significantly higher prevalence of functional high-risk criteria in the older group (23, 25, 26). Just like our patient population, Wintzer-Wehekind et al. and a prospective case study by Poli et al. described no difference regarding the distribution of patients with high-risk criteria between the groups (22, 27). Accordingly, the DEFENSE-PFO trial only included only patients with high-risk morphologies defined as defect size ≥2 mm or ASA with an excursion between 10 and 15 mm (21). These authors concluded from a subgroup analysis that PFO occlusion may be especially beneficial in patients above the age of 60 years (28). ASA and PFO features, such as defect size and shunt volume, are known risk factors for stroke recurrences (29, 30), and patients with high-risk shunts or ASA may have a greater benefit from PFO occlusion, compared with patients with small defects (31). Likewise, Giacoppo et al. showed in a large meta-analysis that the number needed to treat to prevent one recurrent ischemic event was 13 in patients with high-risk shunt features compared with 24 in unselected patients (32).

### Outcome and safety in older vs. younger patients

The rate of unsuccessful implantation was low, with one case in each group. Repositioning or change of the initially used device size or type had to be performed in 4.8% of the cases, with no difference between the age groups. Post-interventional, no relevant protrusion or dislocation could be seen. Residual shunting was detected in 5.3% of the younger patients vs. 6.3% in the older patients (*p* = 1). Access site complications occurred in 2.7% of the total population. One air embolism happened during the implantation and required interventional treatment. One pericardial tamponade requiring percutaneous drainage occurred during the inpatient stay. Both patients were discharged without impairment after treatment. There was one in-hospital death in each group due to septic organ failure, without association with the procedure. During the 6-month follow-up, residual shunts were discovered in 16.1 vs. 11.3% of the cases (*p* = 0.425). Occluder implantation was proven to be safe without a device or intervention-related death within the observation time of 6 months after hospital discharge. Four thromboembolic events occurred during the 6-month follow-up. One in the younger group and three in the older group, representing a rate of 2.8% in the total study population (1.6% in the younger and 3.8% in the older group (*p* = 0.817). One death occurred in the elderly age group during long-term follow-up 7 months after implantation due to the progression of cancer.

The rate of patients with residual shunts was minimally higher than described by Luersmann et al. (26) with 7.7% in the younger and 12.1% in the older age group. We confirmed an overall low rate of recurrence of thromboembolic events, as described by Luersmann et al. and Alperi et al. (23, 26). Still, we could not replicate a significantly higher rate of events in the older age group in our population with balanced baseline criteria regarding shunt parameters and a low rate of pre-known atrial fibrillation. Findings were in line with the publications by Spies et al. and Wintzer-Wehekind et al., who also observed comparable recurrence rates of thromboembolic events across both age groups, especially after adjustment for baseline cardiovascular risk factors (22, 25). Yet, Wintzer-Wehekind et al. reported a higher incidence of new-onset atrial fibrillation compared with our observations (22).

### Outcome in high- vs. low-volume shunts

To evaluate the impact of anatomical and functional parameters of the PFO on the outcome in elderly patients, we subdivided the cohort according to high-volume criteria (grade 3 shunt and/or defect size ≥10 mm). Groups had balanced baseline criteria. However, larger shunts were associated with more complex defects and a larger left atrial size as a sign of high-volume right-to-left shunting. Both groups showed a low event rate and no difference in procedural parameters such as procedure duration, peri-interventional complications, rate of unsuccessful implantation attempts or need for repositioning, and outcome parameters, such as recurrence of thromboembolic complications or new-onset atrial fibrillation, long-term complications, or death. The trend toward a higher rate of residual shunts in the HSV group was statistically not significant. Per definition in our analysis, ASA was not included as a parameter defining HVS and was equally distributed between both groups. A large, pooled analysis of 6 RCTs published by Mas et al. has shown an impact of larger PFO size (shunt size ≥20–30 bubbles) on outcome in a cohort with a mean age of 45 years with a risk reduction of 2% compared to 1% and a number needed to treat of 49, instead of 98 in a cohort with small PFO (31). This effect could not be reproduced in our elderly cohort.

Our findings support data that have shown that intra-atrial shunt occlusion is a suitable and safe option even for elderly patients after ESUS with a high procedural success and a low event rate. Our data underline the finding that a benefit may also be derived when treating patients who do not have functional high-risk constellations.

### Limitations

There are important limitations of this study. The current study was designed as a single-center, retrospective observational study. Therefore, this study relies on the analysis of existing medical records, introducing potential biases and limitations related to the collection and availability of data. Furthermore, the small number of included patients leads to few events in total and a low statistical power to find small differences between the study groups. Furthermore, compliance with follow-up was low with participation of only 75.9% of the patients. Also, a follow-up of 6 months does not allow us to analyze the long-term benefit of PFO closure. Long-term follow-up analysis was restricted to patients who experienced readmissions to our institution. This introduces a selection bias, as it disproportionately includes data from patients experiencing complications, who are more likely to require readmission. Consequently, the long-term outcomes reported may not be fully representative of the entire study cohort.

## Conclusion

Occlusion of intra-atrial shunts is a safe and effective option for secondary prevention of ESUS in patients aged >50 years. The outcome in this elderly cohort seems to be independent of a functional or anatomic high-risk constellation. This finding needs to be confirmed in randomized controlled trials.

## Data Availability

The original contributions presented in the study are included in the article/Supplementary Material, further inquiries can be directed to the corresponding author.
